# Esophageal Perforation Caused by an Ingested Fish Bone Leading to Aortic Pseudoaneurysm: A Rare Vascular Complication in a 54-Year-Old Patient

**DOI:** 10.7759/cureus.103083

**Published:** 2026-02-06

**Authors:** Martin Nguyen, Tai Nguyen, Long Nguyen, Thai Nguyen, Thao Pham

**Affiliations:** 1 Radiology, West Virginia School of Osteopathic Medicine, Lewisburg, USA; 2 Obstetrics and Gynecology, Mekong Hospital, Ho Chi Minh, VNM; 3 Obstetrics and Gynecology, Hung Vuong Hospital, Ho Chi Minh, VNM; 4 Internal Medicine, District 3 General Hospital, Ho Chi Minh, VNM; 5 Radiology, University Medical Center, Ho Chi Minh, VNM

**Keywords:** aortic pseudoaneurysm, aortoesophageal fistula, esophageal foreign bodies, esophageal perforation, fish bone

## Abstract

Ingestion of sharp esophageal foreign bodies (EFBs) such as fish bones is common and usually benign, but transmural perforation can lead to rare, life-threatening vascular complications. A 54-year-old woman presented with acute chest pain after fish bone ingestion. Computed tomography angiography (CTA) demonstrated a linear radiopaque FB penetrating the esophageal wall at the T3 level and located near the proximal descending thoracic aorta. Endoscopic removal confirmed esophageal perforation, and broad-spectrum intravenous antibiotics were initiated. Despite initial management, follow-up imaging revealed the development of a thoracic aortic pseudoaneurysm (AP) at the site of penetration, prompting urgent thoracic endovascular aortic repair (TEVAR) with adjunctive hybrid surgical measures to preserve cerebral perfusion. The postoperative course was uneventful, and subsequent imaging showed durable stent patency without evidence of infection or rupture. This case highlights the potential for delayed vascular injury following esophageal perforation, even after successful FB removal, and underscores the importance of close imaging surveillance, aggressive infection control, and coordinated multidisciplinary management to prevent progression to catastrophic aortoesophageal fistula (AEF).

## Introduction

In the USA, esophageal foreign bodies (EFBs) occur in about 100,000 cases annually [1]. Accidental FB ingestion in adults often occurs in the elderly with cognitive impairment, while intentional ingestion may happen in individuals with psychiatric disorders [2]. The typical presentation includes acute dysphagia and the inability to swallow saliva. Other symptoms may include odynophagia, retrosternal pain, FB sensation, vomiting, and drooling [1]. Approximately 1%-3% of patients require surgery due to complications, including perforation, irretrievable FB, mediastinitis, empyema, fistula, and severe hemorrhage [3-5]. Osseous FB, such as fish or chicken bones, is a leading cause of esophageal injury [6].

Although the majority of ingested FB passes spontaneously, surgical intervention is required in less than 1% of cases [7]. In a landmark review of over 2,000 cases, aortic injury occurred in <0.1% of patients [8]. Within this rare subset, aortic pseudoaneurysm (AP) serves as a precursor to the more severe stage of aortoesophageal fistula (AEF). Because the mortality of untreated AEF approaches 100% [9], the identification of a pseudoaneurysm in a hemodynamically stable patient is one of the most important factors for survival. Here, we present an unusual case of pseudoaneurysm of the aorta caused by a fish bone in a middle-aged woman, which was successfully surgically managed.

## Case presentation

A 54-year-old woman presented to the emergency department with acute chest pain following ingestion of a fish bone. She reported accidentally swallowing a bone during a meal, which became lodged in her throat. Attempts to dislodge it by swallowing rice were unsuccessful, leading to persistent throat discomfort and subsequent midline chest pain exacerbated by forceful coughing, without radiation or associated factors. She denied fever, dyspnea, hoarseness, odynophagia, or gastrointestinal bleeding.

Her past medical history was unremarkable, with no chronic illnesses, prior surgeries, or known allergies to medications or foods. She was not taking any regular medications and reported no tobacco, alcohol, or recreational drug use.

**Figure 1:**
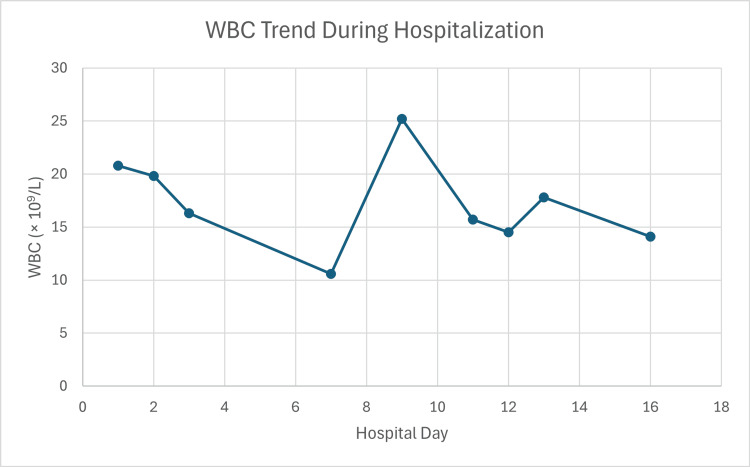
Trend of white blood cell count (×109/L) during hospitalization

On examination, the patient was alert, tachycardic (115 bpm), and mildly febrile (37.9°C). Vital signs were otherwise stable, with normal blood pressure (120/80 mmHg), respiratory rate (18/min), and oxygen saturation (95%). Physical findings included a supple neck without masses or lymphadenopathy, regular cardiac rhythm without murmurs, clear lung fields, and a soft, nontender abdomen. No focal neurological deficits were noted. Laboratory investigations revealed marked leukocytosis (20.8 × 10^9^/L) with neutrophilia (89.9%) (Figure 1), elevated C-reactive protein (43.1 mg/L), consistent with an inflammatory or infectious process (Table 1).

**Table 1:** Pertinent laboratory findings on initial presentation CRP: C-reactive protein; Hb: hemoglobin; Hct: hematocrit; MCH: mean corpuscular hemoglobin; MCV: mean corpuscular volume; PLT: platelet; WBC: white blood cell

Lab	Result	Normal range
WBC	20.8 × 10^9^/L	4-11 × 10^9^/L
Neutrophils (%)	89.9%	40-70%
Hb	144 g/L	129-160 g/L
Hct	42.7%	36-47%
MCV	87.7 fL	80-95 fL
MCH	29.6 pg	26-34 pg
PLT	401 × 10^9^/L	150-400 × 10^9^/L
CRP	43.1 mg/L	<1.5 mg/L

**Figure 2:**
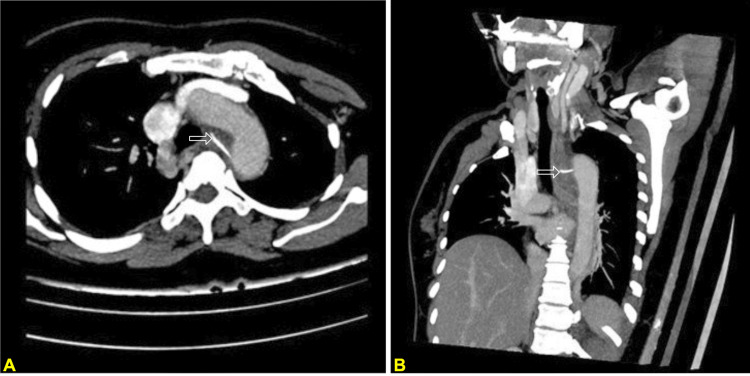
CT scan demonstrated a linear radiopaque foreign body (#2.3 cm), consistent with a fish bone (white arrows) CT: computed tomography The object was located at the level of the T3 vertebra, and the posterior end pointed toward the descending aorta. Associated esophageal wall thickening and periesophageal edema were noted. No definite abscess or pneumomediastinum was demonstrated. (A) Axial plane, (B) coronal plane. White arrows (A and B panels) demonstrated the fish bone

Initial imaging with computed tomography (CT) demonstrated a linear radiopaque FB, consistent with a fish bone approximately 2.3 cm in length, penetrating the esophageal wall at the level of the T3 vertebral body (Figure 2). The posterior tip abutted the wall of the proximal descending thoracic aorta, with associated esophageal wall thickening, periesophageal edema, but no definite abscess or pneumomediastinum.

**Figure 3:**
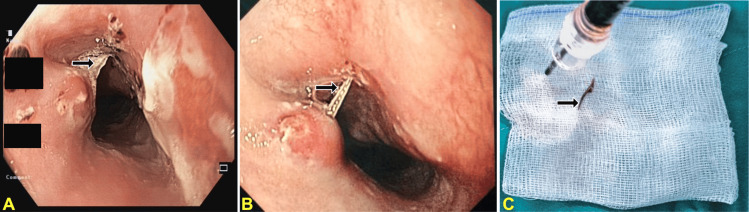
EGD demonstrated a fish bone (black arrows in (A) and (B)) perforating esophageal wall about 20 cm from the incisors. It was successfully removed endoscopically (C) without any complications EGD: esophagogastroduodenoscopy (A) and (B) Fish bone was visualized from different angles, and inflammation of the surrounding mucosa was noted. (C) Fish bone after removal. Black arrows (A, B, and C panels) demonstrated the fish bone

**Figure 4:**
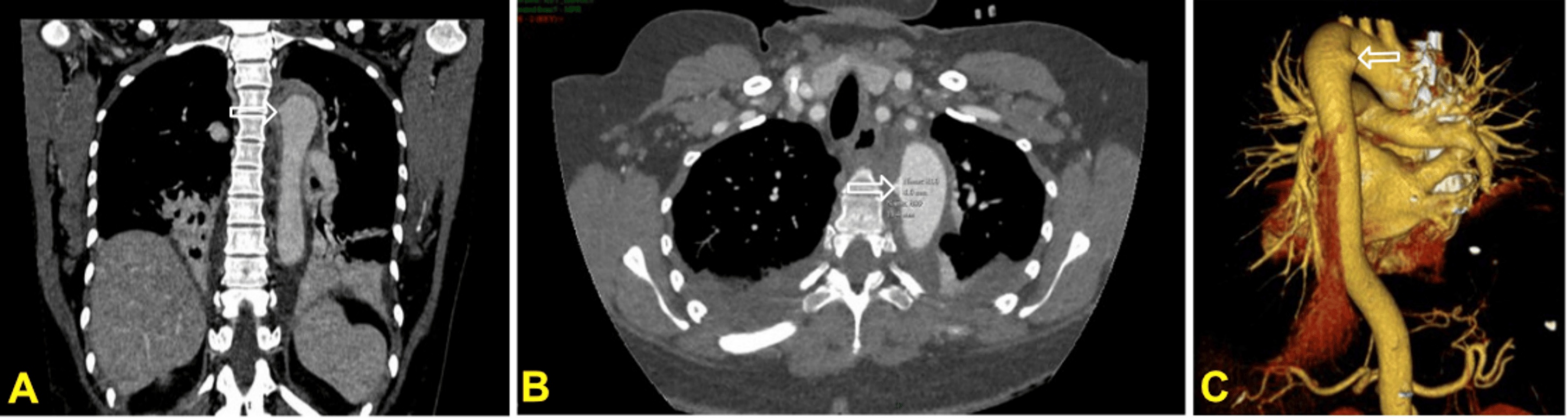
CT scan demonstrated a pseudoaneurysm (white arrows) at the previous location of the fish bone (at the level of T3 vertebra), measuring 4 mm × 10 mm CT: computed tomography; CTA: computed tomography angiography Mild bilateral pleural effusion was noted. (A) and (B), coronal and axial planes; (C), CTA volume-rendered reconstruction. The white arrow (A, B, and C panels) demonstrated the aortic pseudoaneurysm

Esophagogastroduodenoscopy confirmed the fish bone perforating the esophagus at approximately 20 cm from the incisors, with surrounding mucosal inflammation and edema (Figure 3). The bone was successfully removed endoscopically without complication. Mild antral gastritis was noted incidentally. Afterward, she was treated with broad-spectrum intravenous antibiotics (ertapenem and vancomycin) for suspected mediastinitis. Routine follow-up imaging (four days later) showed resolution of the FB but development of a small pseudoaneurysm (depth 4 mm, length 10 mm) in the proximal descending thoracic aorta at the site of prior penetration (Figure 4), with persistent mediastinal inflammation, periaortic fluid, and enhancing plaques indicative of inflammation.

Given the high risk of rupture, the patient underwent urgent thoracic endovascular aortic repair (TEVAR) with deployment of a covered stent-graft to exclude the pseudoaneurysm. Intraoperative angiography revealed unintentional partial coverage of the left common carotid artery origin by the proximal stent edge. To preserve cerebral perfusion, immediate total aortic arch debranching was performed using an 8 mm silver-coated synthetic graft. This involved end-to-side anastomosis of the graft to the right common carotid artery and to the left common carotid artery, with proximal ligation of the left common carotid near its aortic origin and creation of a left carotid-subclavian bypass. Intraoperative cerebral oximetry remained stable throughout.

**Figure 5:**
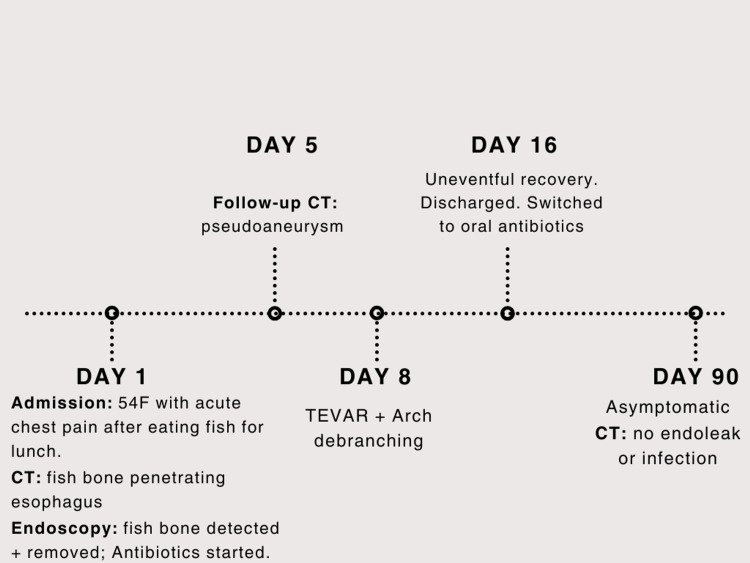
Important milestones during patient management CT: computed tomography; TEVAR: thoracic endovascular aortic repair

The postoperative course was uncomplicated, with resolution of symptoms and no neurological sequelae. Subsequent imaging confirmed stent-graft patency, stable mediastinal changes, and a small type II endoleak without evidence of ongoing infection or rupture. She was discharged after about two weeks. Leukocyte count decreased significantly at this time (from 20.8 × 10^9^/L on admission to 14.1 × 10^9^/L at day 16). She was transitioned to prolonged oral antibiotic therapy for a total duration of four weeks. At follow-up three months later, the patient remained asymptomatic with excellent functional recovery (Figure 5).

## Discussion

EFBs, particularly fish bones, are common in Asian countries due to dietary habits involving frequent fish consumption [10,11]. In a retrospective study including 427 cases of EFB in China, Ruan reported that 46.4% of cases were due to fish bones and 26.5% to poultry bones [12]. EFBs represent a relatively common presentation in emergency departments, often manifesting as acute dysphagia, inability to swallow saliva, or retrosternal pain [1,12]. EFBs may occur at all levels of the hypopharynx or in the upper portion of the esophagus, potentially due to the lower pressure in the transition zone between striated and smooth muscle fibers [3]. Sharp and pointed objects, such as fish bones, may penetrate the esophageal wall, leading to serious complications, including cervical abscess, mediastinitis, pneumonia, pneumothorax, tracheoesophageal fistula, and, rarely, major vascular injuries such as AEF or AP [7,13,14]. Therefore, when EFBs, especially sharp objects, are suspected, prompt endoscopic evaluation and removal are recommended to minimize complications, morbidity, and mortality [15].

Evaluation of a patient with suspected EFBs should include careful history taking and physical examination [5,16]. The European Society of Gastrointestinal Endoscopy (ESGE) recommended plain radiography for suspected radiopaque objects or when the type of FBs is unknown [16]. A CT scan is recommended in cases of suspected perforation or other complications, which may require surgery [16]. Many swallowed FBs may appear radiolucent on plain radiographs, including fish and chicken bones, plastic, and wood [17]. In these cases, secondary radiographic signs such as prevertebral cervical swelling on a lateral cervical X-ray can suggest the presence of FB in an appropriate setting if it’s consistent with the history [17]. CT scan has been shown to have high sensitivity and specificity (99.8% and 100%) to detect FBs in the upper GI tract, as well as in the detection of fish bones [18,19]. It was also demonstrated to be superior to plain X-rays (sensitivity and specificity of 51.2% and 99.5%, respectively) [19]. Thus, CT scan is considered the first-line imaging modality for radiolucent FBs and a second-line modality when plain X-rays are negative for radiopaque cases; however, clinical suspicion remains high for impacted FBs [19]. 

The diagnostic algorithm for suspected EFBs has evolved regarding the threshold for cross-sectional imaging, though plain radiography remains the universal initial screen [5,16,20]. While all major guidelines agree that biplane radiography is effective for radiopaque objects, there are some differences in how they manage radiolucent ingestions (Table 2). The 2011 guidelines of the American Society for Gastrointestinal Endoscopy (ASGE) suggested that while CT may be useful, it is not definitive, and persistent symptoms mandate endoscopic evaluation even in the setting of negative imaging [5]. In contrast, the 2016 guidelines of the ESGE provide a more quantitative warning regarding the limitations of plain films, explicitly noting a sensitivity of only 32% for detecting fish bones, and report a CT sensitivity of 90%-100% for these cases [16]. From a radiology standpoint, Guelfguat et al. advocate for the aggressive utilization of CT, citing a sensitivity of up to 100% for sharp FBs such as fish bones. Consequently, the take-home point for clinical practice is that a negative plain X-ray is insufficient to exclude FB impaction due to soft tissue obscuration, especially in cases of high clinical suspicion [20]. Current practice increasingly aligns with this radiological perspective, favoring early CT utilization to prevent missed diagnoses of these types of FBs [20].

**Table 2:** Guideline recommendations for diagnostic imaging of esophageal foreign bodies ^1^ASGE: American Society for Gastrointestinal Endoscopy; ^2^ESGE: European Society of Gastrointestinal Endoscopy; ^3^AJR: American Journal of Roentgenology

Clinical parameter	ASGE^1^ guidelines (2011) [5]	ESGE^2^ guidelines (2016) [16]	AJR^3^ guidelines (2014) [20]
Primary screening modality	Biplane radiography: recommended to confirm the location, size, and shape of radiopaque objects and exclude free mediastinal air	Plain radiography: recommended to assess radiopaque objects^5^. Not recommended for uncomplicated nonbony food bolus impaction	Plain radiography: an initial screening tool, but acknowledged to have significant limitations for radiolucent objects
Sensitivity for fish bones	Low/variable: notes that fish or chicken bones are "not readily seen" on plain films^8^	Quantified low (32%): explicitly cites prospective data indicating plain radiography has a sensitivity of only 32% for fish bones^9^	Poor: states radiography "poorly visualizes fish bones" due to variable calcification and obscuration by soft tissue masses^10^
Indications for CT	Adjunctive/limited: "may be useful" if radiographs are negative, though notes CT may still fail to detect radiolucent objects^11^	Complication-focused: strongly recommended for patients with suspected perforation or complications requiring surgery	Diagnostic standard: considered the "test of choice" for fish bone impaction. Sensitivity reported at 90-100% with superior detection of "invisible" objects compared to plain film
Use of contrast studies	Contraindicated: generally should not be performed due to aspiration risk and interference with subsequent endoscopic visualization	Contraindicated (barium): barium swallow is not recommended. Water-soluble contrast is considered only if the X-ray is negative and obstruction is not suspected	Restricted: use of oral contrast is controversial and often discouraged due to aspiration risk. Water-soluble agents are reserved for confirming suspected perforation
Management of negative imaging	Endoscopy mandatory: persistent esophageal symptoms require endoscopic evaluation even in the setting of negative radiographic evaluation	Endoscopy mandatory: if X-ray is negative but suspicion remains, diagnostic work-up should proceed; medical treatment (e.g., glucagon) should not delay endoscopy	Advanced imaging (CT): a negative radiograph is insufficient to rule out sharp radiolucent objects; CT is indicated to unmask complications occult on plain film
Key distinction	Views CT primarily as a problem-solving tool when plain films are equivocal	Establishes the specific 32% sensitivity statistic for plain films, justifying a lower threshold for advanced imaging	Advocates for CT with 3D reconstruction as the definitive modality for radiolucent foreign bodies (fish bones, wood, plastic)

Historically, AP and AEF secondary to FB ingestion carried a high mortality rate under conservative management, largely due to rapid progression to exsanguination and mediastinal sepsis [9,21]. AEF is an extremely rare entity in a scenario of ingested FBs, with the incidence of less than 0.1% [8]. In a study of 2394 cases of ingested FBs by Nandi et al. [8], only two cases of AEF were reported. In another study of 1338 cases of FBs conducted by Lai et al. [22], none was reported to have AEF.

Regarding treatment, two main approaches include endoscopy and surgery. However, 90% of all cases of ingested FBs will pass spontaneously. About 10%-20% of those FBs can be removed endoscopically, and 1% of all cases require surgery [23]. Endoscopy is the main intervention to remove EFBs, including both flexible endoscopy (FE) and rigid endoscopy (RE). FE is currently considered first-line therapy in the majority of EFB cases, while RE may be more suitable in a few selected cases [24]. Surgery is considered one of the last resorts when severe complications are suspected [12].

For AEF, open surgical repair (OSR) has a high mortality rate (55.5%) even in tertiary care centers [25]. Recently, TEVAR gained popularity as an emergent treatment for AEF [21]. A recent systematic review suggested TEVAR achieved a high technical success rate (87.3%) with a 30-day mortality rate of 19.4% [21]. In acute settings, TEVAR helped control bleeding and stabilize hemodynamics, thus also improved morbidity and mortality associated with OSR [21]. However, TEVAR alone will not treat an esophageal defect, and the stent graft would be exposed to a contaminated environment [21]. AEF recurrence and stent graft infection were 13.8% and 15.2%, respectively [21]. In a systematic review of 55 articles including 72 patients with AEF treated with TEVAR, Canaud et al. [21] reported that all-cause mortality was 40.2% at 7.4 months of follow-up. Prolonged antibiotic use (>4 weeks) was utilized in 80% of patients, and it was the only factor that was associated with a significantly lower incidence of aortic mortality in the multivariate analysis [21]. Due to the high risk of endograft infection and mediastinitis after TEVAR [21,26], some authors considered it as a bridging therapy for patient stabilization before definitive aortic surgery combined with esophageal or bronchial repair [27,28]. In summary, TEVAR is rarely used as a stand-alone procedure, stent-graft removal can be considered, and the esophageal lesion can be surgically addressed once the patient's condition has stabilized [26,29]. In a case of AEF caused by a chicken bone, Chen et al. [30] proposed an alternative approach including an aortic stent graft placement combined with thoracoscopic mediastinal debridement and drainage, as well as an esophageal stent placement to isolate the fistula. After 80 days, the esophageal stent was removed [30]. In this paper, we also reviewed previously reported cases of EFBs complicated by AP or AEF that were managed with endovascular stent treatment over the past two decades (2005-2026) (Table 3).

**Table 3:** Clinical characteristics, treatment strategies, and outcomes of patients treated with TEVAR for aortic injury secondary to esophageal foreign body ingestion, including cases of pseudoaneurysm and aortoesophageal fistula (2005-2026) AEF: aortoesophageal fistula; mo: month; TEVAR: thoracic endovascular aortic repair; WBC: white blood cell; MRSA: Methicillin-resistant *Staphylococcus aureus* Note: "Treatment failure" is defined as death or need for rescue open surgery

Author (year)	Age/sex	Foreign body	Diagnosis	Treatment strategy	Infection/complications	Outcome
Ma et al. (2026) [31]	74 F	Fish bone	Ruptured Pseudoaneurysm	TEVAR + endoscopic clips	Persistent mediastinitis	Recurrent bleeding (treatment failure)
Gong et al. (2022) [32]-case 1	71 F	Fish bone	Aortic perforation/AEF	TEVAR (no open surgery)	None	Survived (alive at 19 mo)
Gong et al. (2022) [32]-case 2	48 M	Fish bone	Aortic impalement	Simultaneous endoscopy + TEVAR	Transient fever	Survived (alive at 5 mo)
Zeng et al. (2020) [33]	58 M	Duck bone	Intramural hematoma	TEVAR + endoscopic removal	Not reported	Survived
Rawala et al. (2018) [34]	80 F	Unknown	AEF + aneurysm	TEVAR only	MRSA sepsis (stent infection)	Died (3 mo postop)
Shen et al. (2018) [35]	40 M	Chicken bone	AEF + pseudoaneurysm	TEVAR + thoracotomy (omentum flap)	Mild leukocytosis	Survived
Mezzetto et al. (2016) [27]	79 M	Goat bone	Pseudoaneurysm	TEVAR + endoscopic clips	Fever + leukocytosis	Survived
Xi et al. (2013) [28]	25 M	Fish bone	Pseudoaneurysm	TEVAR only	None	Survived
Chen et al. (2012) [30]	22 M	Chicken bone	AEF + pseudoaneurysm	TEVAR + thoracoscopy (debridement)	Leukocytosis (WBC 19.9)	Survived
Kelly et al. (2009) [9]	59 M	Fish bone	AEF	TEVAR → explantation	Sepsis (stent infection)	Survived (required open repair)
Assink et al. (2005) [36]	32 M	Fish bone	AEF	TEVAR + thoracotomy	None	Survived

Mediastinal infection seemed to be the primary predictor of increased mortality and failure rate of TEVAR (Table 3). This is consistent with the study of Canaud et al. [21] in which the authors reported that prolonged antibiotic use was the only factor associated with a lower incidence of aortic mortality (p = 0.003). The analysis of TEVAR outcomes in this setting reveals significantly different outcomes based on the presence of infection (Table 3). In cases where deep mediastinal infection was present and not aggressively managed, TEVAR frequently served only as a temporary seal before eventual catastrophic failure. For instance, Rawala et al. [34] reported a patient who died three months post-procedure due to sepsis from an infected endovascular stent, and Kelly et al. [9] described a case where the stent became infected 51 days after insertion, necessitating high-risk explantation and definitive open surgical reconstruction. An endograft placed in an infected field acts as an FB itself; without adequate debridement, it can become a nidus for persistent infection, leading to pseudoaneurysm recurrence or fistula reformation. This failure mode was recently reinforced by Ma et al. [31], who reported treatment failure due to persistent mediastinitis despite initial sealing of TEVAR.

## Conclusions

EFB is a common emergency presentation but can rarely lead to catastrophic aortic complications such as pseudoaneurysm and AEF. Early endoscopic evaluation combined with prompt cross-sectional imaging is critical for detecting esophageal perforation and identifying evolving vascular injury. This case highlights the importance of serial imaging, meticulous surgical planning, and comprehensive postoperative management in the treatment of AP secondary to FB ingestion. Current evidence supports a hybrid approach using TEVAR as initial stabilization, followed by staged definitive management when feasible, with aggressive and prolonged antibiotic therapy to control mediastinal infection. Close clinical and imaging surveillance remains essential for early detection of endograft-related complications and prompt interventions.

## References

[1] Chirica M, Kelly MD, Siboni S (2019). Esophageal emergencies: WSES guidelines. World J Emerg Surg.

[2] Wyllie R (2006). Foreign bodies in the gastrointestinal tract. Curr Opin Pediatr.

[3] Aiolfi A, Ferrari D, Riva CG, Toti F, Bonitta G, Bonavina L (2018). Esophageal foreign bodies in adults: systematic review of the literature. Scand J Gastroenterol.

[4] Athanassiadi K, Gerazounis M, Metaxas E, Kalantzi N (2002). Management of esophageal foreign bodies: a retrospective review of 400 cases. Eur J Cardio-Thorac Surg Off J Eur Assoc Cardio-Thorac Surg.

[5] Ikenberry SO, Jue TL, Anderson MA (2011). Management of ingested foreign bodies and food impactions. Gastrointest Endosc.

[6] Xiong J, Cao J, Yu J, Li P, Zeng Z, Pan X (2025). Case report: area of focus in a case of giant aortic arch pseudoaneurysm following fish bone penetration. Front Cardiovasc Med.

[7] Ambe P, Weber SA, Schauer M, Knoefel WT (2012). Swallowed foreign bodies in adults. Dtsch Arztebl Int.

[8] Nandi P, Ong GB (1978). Foreign body in the oesophagus: review of 2394 cases. Br J Surg.

[9] Kelly SL, Peters P, Ogg MJ, Li A, Smithers BM (2009). Successful management of an aortoesophageal fistula caused by a fish bone-case report and review of literature. J Cardiothorac Surg.

[10] Wang A, Zhou Y, Huang Q (2019). A fish bone induced aortic arch pseudoaneurysm in a male patient: a case report. Medicine (Baltimore).

[11] Arulanandam S, Das De S, Kanagalingam J (2015). A prospective study of epidemiological risk factors for ingestion of fish bones in Singapore. Singapore Med J.

[12] Ruan WS, Li YN, Feng MX, Lu YQ (2020). Retrospective observational analysis of esophageal foreign bodies: a novel characterization based on shape. Sci Rep.

[13] Peng A, Li Y, Xiao Z, Wu W (2012). Study of clinical treatment of esophageal foreign body-induced esophageal perforation with lethal complications. Eur Arch Otorhinolaryngol.

[14] Zhao XH, Lu YQ (2014). Multiple embolisms resulted from a huge fishbone piercing the left atrium. Intensive Care Med.

[15] Zhang X, Jiang Y, Fu T, Zhang X, Li N, Tu C (2017). Esophageal foreign bodies in adults with different durations of time from ingestion to effective treatment. J Int Med Res.

[16] Birk M, Bauerfeind P, Deprez PH (2016). Removal of foreign bodies in the upper gastrointestinal tract in adults: European Society of Gastrointestinal Endoscopy (ESGE) Clinical Guideline. Endoscopy.

[17] Tseng HJ, Hanna TN, Shuaib W, Aized M, Khosa F, Linnau KF (2015). Imaging foreign bodies: ingested, aspirated, and inserted. Ann Emerg Med.

[18] Watanabe K, Kikuchi T, Katori Y, Fujiwara H, Sugita R, Takasaka T, Hashimoto S (1998). The usefulness of computed tomography in the diagnosis of impacted fish bones in the oesophagus. J Laryngol Otol.

[19] Huang H, Choi RC, Biswas RK, Katelaris P, Ridley L (2026). Meta-analysis comparing the diagnostic accuracy of X-rays and CT in detecting ingested foreign bodies in the neck. J Med Imaging Radiat Oncol.

[20] Guelfguat M, Kaplinskiy V, Reddy SH, DiPoce J (2014). Clinical guidelines for imaging and reporting ingested foreign bodies. AJR Am J Roentgenol.

[21] Canaud L, Ozdemir BA, Bee WW, Bahia S, Holt P, Thompson M (2014). Thoracic endovascular aortic repair in management of aortoesophageal fistulas. J Vasc Surg.

[22] Lai H, Ge D, Zheng YJ, Li J, Wang C (2011). Surgical management of aortoesophageal fistula caused by foreign bodies. Eur J Cardiothorac Surg.

[23] Webb WA (1995). Management of foreign bodies of the upper gastrointestinal tract: update. Gastrointest Endosc.

[24] Gmeiner D, von Rahden BH, Meco C, Hutter J, Oberascher G, Stein HJ (2007). Flexible versus rigid endoscopy for treatment of foreign body impaction in the esophagus. Surg Endosc.

[25] Kieffer E, Chiche L, Gomes D (2003). Aortoesophageal fistula: value of in situ aortic allograft replacement. Ann Surg.

[26] Marone EM, Coppi G, Kahlberg A, Tshomba Y, Chiesa R (2010). Combined endovascular and surgical treatment of primary aortoesophageal fistula. Tex Heart Inst J.

[27] Mezzetto L, Treppiedi E, Scorsone L, Giacopuzzi S, Perandini S, Macrì M, Veraldi GF (2016). Thoracic aortic pseudoaneurysm after esophageal perforation and mediastinitis caused by accidental ingestion of a mutton bone: a case report on staged endoscopic and endovascular treatments. Ann Vasc Surg.

[28] Xi EP, Zhu J, Zhu SB (2013). Surgical treatment of aortoesophageal fistula induced by a foreign body in the esophagus: 40 years of experience at a single hospital. Surg Endosc.

[29] Topel I, Stehr A, Steinbauer MG, Piso P, Schlitt HJ, Kasprzak PM (2007). Surgical strategy in aortoesophageal fistulae: endovascular stentgrafts and in situ repair of the aorta with cryopreserved homografts. Ann Surg.

[30] Chen X, Li J, Chen J (2012). A combined minimally invasive approach for the treatment of aortoesophageal fistula caused by the ingestion of a chicken bone: case report and literature review. Clinics (Sao Paulo).

[31] Ma Y, Zhang L, Ji F (2025). Case report: aortoesophageal fistula induced by a fish bone: the critical role of mediastinal infection control after TEVAR and endoscopic closure. Front Cardiovasc Med.

[32] Gong H, Wei W, Huang Z, Hu Y, Liu XL, Hu Z (2022). Endovascular stent-graft treatment for aortoesophageal fistula induced by an esophageal fishbone: two cases report. World J Clin Cases.

[33] Zeng L, Shu W, Ma H, Hu J (2020). Aortic injury caused by esophageal foreign body-case reports of 3 patients and literature review. Medicine (Baltimore).

[34] Rawala MS, Badami V, Rizvi SB, Nanjundappa A (2018). Aortoesophageal fistula: a fatal complication of thoracic endovascular aortic stent-graft placement. Am J Case Rep.

[35] Shen JY, Zhang HW, Fan KJ, Liao H, Zhang EY, Hu J (2018). Aortoesophageal fistula and arch pseudoaneurysm after removing of a swallowed chicken bone: a case report of one-stage hybrid treatment. BMC Surg.

[36] Assink J, Vierhout BP, Snellen JP, Benner PM, Paul MA, Cuesta MA, Wisselink W (2005). Emergency endovascular repair of an aortoesophageal fistula caused by a foreign body. J Endovasc Ther.

